# Severe Myocarditis in a Female Following mRNA-1273 Vaccine: A Case Report and Review of the Literature

**DOI:** 10.7759/cureus.29299

**Published:** 2022-09-18

**Authors:** Bara M AL-Qudah, ELMustafa Abdalla, Fatima Albazoon, Mhd Baraa Habib, Abdel-Naser Y Elzouki

**Affiliations:** 1 Internal Medicine, Hamad Medical Corporation, Doha, QAT; 2 Medicine, Weill Cornell Medical College, Doha, QAT

**Keywords:** covid-19 virus vaccine-related myocarditis, vaccine-associated myocarditis, mrna vaccine myocarditis, covid-19 vaccine, covid-19

## Abstract

Myocarditis was recently described as one of the complications secondary to COVID-19 vaccination. We present a 38-year-old lady diagnosed with vaccine-related myocarditis a few days after receiving the mRNA-1273 vaccine. We also summarize what is reported in the literature about the association between COVID-19 vaccination and myocarditis. In conclusion, COVID-19 immunization appears to be associated with significantly fewer adverse outcomes than COVID-19 infection among all age groups.

## Introduction

Myocarditis is inflammation of cardiac muscles, which can be diffuse or focal in involvement, acute, subacute, or chronic in the clinical course. Multiple infectious and non-infectious etiologies have been identified [1].

Viral (idiopathic) myocarditis is the most commonly recognized cause so far, adenovirus, coxsackievirus, and enteroviruses are well-identified culprits, and recently many COVID infection-induced myocarditis cases have been reported [2]. Exposure to non-infectious agents, including medications causing toxic myocarditis and post-vaccinated myocarditis like influenza and recently mRNA COVID-19 vaccine, is also being reported [3,4].

The pathophysiology of myocarditis can be due to the direct toxic effect of the offending agent or secondary autoimmune response and cytokine activation in the myocardium. In vaccine-induced myocarditis, autoimmune response due to molecular mimicry and cytokines activation mostly play a significant role in the disease pathogenesis [5,6]. The clinical presentation of acute myocarditis varies; some patients present with acute coronary syndrome (ACS), with symptoms such as chest pain, electrocardiogram (ECG) changes, and high troponin, and others present with new or worsening heart failure or ventricular arrhythmias. Clinical suspicion of myocarditis is increased in patients with cardiac symptoms, elevated troponin, and ECG changes with a lack of risk factors for ACS [1,7].

In addition to clinical history, examination, troponin level, and ECG, imaging for diagnosing myocarditis includes an echocardiogram to assess left ventricle (LV) dilation and focal or global regional wall motion abnormalities. Moreover, coronary angiography, which is done in selected patients to rule out ACS, and cardiac MRI where the presence of edema, hyperemia, and scarring, further support the diagnosis of myocarditis. Although endocardial biopsy is rarely indicated, it can be considered when the results affect the management approach [1,7].

## Case presentation

A 38-year-old female patient from Jordan with a known case of asthma presented to the emergency department (ED) on May 22, 2021, due to 5 days history of fever (38.5-39°C) associated with chills, rigors, dry cough, shortness of breath, and neck pain with mild chest discomfort. The patient’s history was negative for flu, sore throat, headache, photophobia, gastrointestinal (GI) symptoms, ear pain, or discharge. The patient received mRNA-1273 (Moderna) vaccine first dose on March 26, 2021, confirmed COVID positive case on April 27 and received mRNA-1273 (Moderna) vaccine second shot on May 12, 2021. The patient was on cloxacillin prescribed from the health center due to mastitis, diagnosed on May 19. The mastitis symptoms were improved already when she got admitted on May 25, 2021.

**Figure 1:**
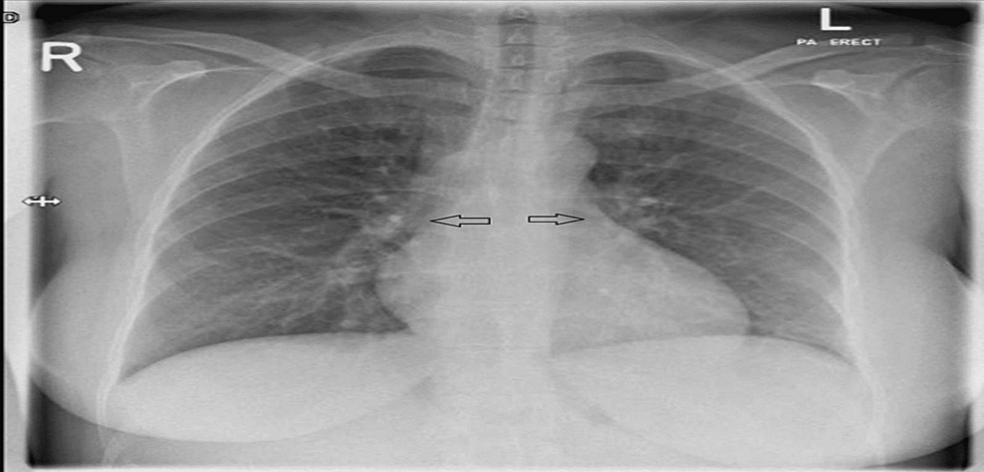
Chest X-ray arrows show bilateral prominent bronchovascular markings with no obvious consolidation or infiltrate seen

Upon presentation to ED, the patient was on room air, tolerating an O2 saturation of 98%, her body temperature was 39°C, her blood pressure was 97/58 mmHg, and her respiratory rate was 20 breaths per minute. The patient’s physical examination and chest were unremarkable. The abdomen on palpation was soft but tender in the right hypochondrium. Cervical lymph nodes were not palpable, but tenderness was positive upon palpation. Based on the presentation and the recent COVID infection pandemic, persistent COVID, pulmonary embolism, cervical lymphadenitis, or sepsis were suspected. The patient was started on antimicrobials (piperacillin/tazobactam & azithromycin). Laboratory workups were sent upon admission (Table 1), and her COVID PCR was negative. A viral panel was done for adenovirus, Epstein-Barr virus (EBV), cytomegalovirus (CMV), HIV, influenza, and other respiratory viruses were negative. A chest X-ray was done on May 23, 2021 (Figure 1).

**Table 1:** Initial laboratory tests WBC: white blood cells, Hgb: hemoglobin, Pro-BNP: pro-brain natriuretic peptide, CRP: C reactive protein

	Value w/Units	Normal Range
WBC	4.0 x 10^3/uL	4.0-10.0
Hgb	9.4 gm/dL	13.0-17.0
Platelet	137 x 10^3/uL	150-400^3
D-dimer	1.99 mg/L	0 to 0.46
Pro-BNP	837 pg/mL	<125
Troponin-T	<3 ng/L	<14
CRP	218 mg/L	0-5

**Figure 2:**
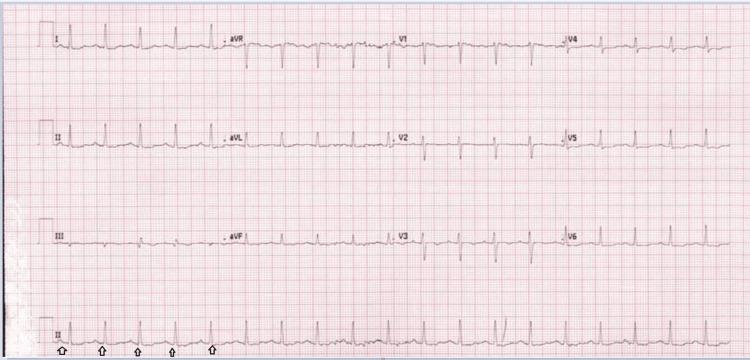
ECG showed sinus tachycardia with nonspecific ST/T wave changes

CT pulmonary angio was done on May 23, 2021, due to the elevated D-dimer level which showed no definite pulmonary embolism. ECG (May 23, 2021) showed sinus tachycardia with no ST segment changes (Figure 2).

**Figure 3:**
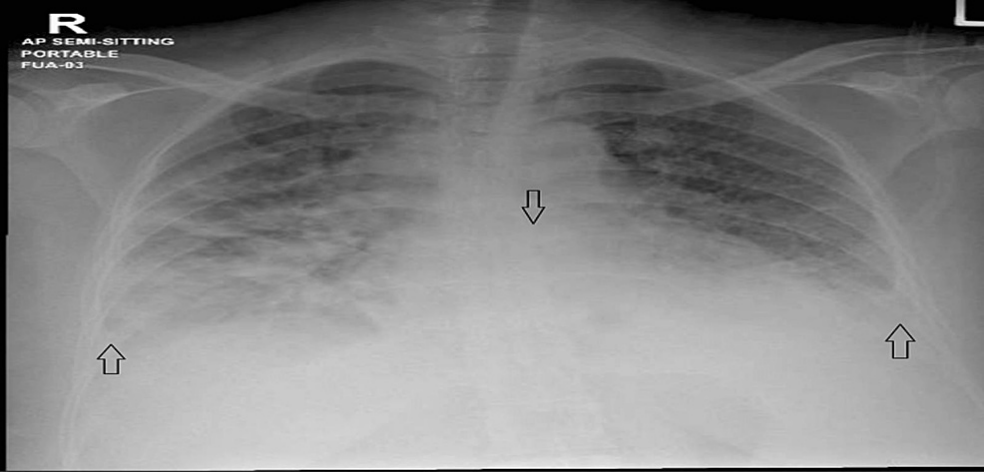
Obliteration of bilateral costophrenic angels with cardiomegaly

Troponin second set sent the next day was elevated at 947 ng/L (reference range 3-10). Echocardiography done on May 24, 2021, showed moderately reduced systolic LV function (ejection fraction (EF) 40%), moderate global hypokinesis of LV, mildly dilated right ventricle (RV), mildly reduced RV function, and mild mitral valve regurgitation present. On May 24, the patient was admitted to the medical intensive care unit, and she was hypotensive (82/63 mmHg), tachycardic, and tachypneic; she was maintaining an oxygen saturation of 95% on a high-flow nasal cannula. IV fluids corrected the patient’s blood pressure; she got overloaded as the chest crepitations were audible and received one dose of IV furosemide 60 mg. The patient received IV dopamine 5 mcg and was kept on piperacillin/tazobactam and azithromycin. The chest X-ray done on May 24 is shown in Figure 3.

The cardiology team reviewed the patient for suspicion of myocardial infarction type II vs. severe myocarditis; she was started on dual antiplatelet therapy (DAPT) with aspirin and clopidogrel and supportive measures. During the intensive care stay from May 24 to May 30, the patient improved gradually, oxygen support weaned off, and blood pressure was on the lower side but off inotropes. Intravenous methylprednisolone 40 mg BID was added on May 27. Blood cultures and urine cultures were negative. On May 30, the patient was clinically stable, felt much better, blood workup parameters significantly improved, and the patient was shifted to the medical ward. The cardiology team introduced bisoprolol 1.25 mg and lisinopril 2.5 mg as blood pressure allowed. Antimicrobials de-escalated to ampicillin sulbactam. Echocardiography was repeated before discharge on (June 2, 2021) and showed normal global systolic LV function (EF 55%) and a minimal pericardial effusion.

She was discharged home after 13 days of hospitalization; she denied any complaints, she was vitally stable and her labs tremendously improved, and her troponin was dropping gradually. Upon discharge, the patient was in stable clinical condition on aspirin, clopidogrel, bisoprolol, and losartan.

Follow up

She was asymptomatic, had no shortness of breath or chest pain, and returned to her baseline status. On the cardiology clinic follow-up, cardiac CT angiography was done on September 19, 2021, and showed a calcium score of zero. And no evidence of coronary artery disease. Laboratory tests showed average complete blood count, renal function tests, and liver enzymes. Troponin-T high sensitivity (4 ng/L, reference range 3-10) and Pro-BNP (41 pg/ml, reference <125 exclude cardiac dysfunction with high certainty) also were completely average values. Aspirin and clopidogrel were stopped and left on bisoprolol only.

## Discussion

The present case had a similar presentation to the recent rare reported myocarditis following the mRNA vaccine; most of the cases described are of the male gender. Our patient had a more severe course; most cases reported except four cases had a mild course and were discharged within 3-6 days of hospitalization [6,8-10]. Also, our patient had low blood pressure readings and a drop in EF with volume overload and hypokinesia in echocardiography. She had elevated troponin, nonspecific ECG changes, and the typical symptoms described in the literature. Thus, cardiac MRI was not necessary for suggesting the diagnosis. She improved with supportive measures and steroids. Other causes of myocarditis were ruled out, COVID PCR was negative twice, and other viruses panel was negative. The echo finding was normalized upon discharge and symptomatically improved with no bacterial source of infection found clinically or in cultures. To date, when writing this case report about the association between mRNA vaccine and myocarditis in adults, we recognized 19 published reports using the PubMed searching database, seven case series, and 12 case reports (Table 2) [4,8-25].

**Table 2:** similar cases reported in PubMed A*: Multi-focal subepicardial late gadolinium enhancement (LGE) was present in 7/7 patients, and additional mid-myocardial LGE was demonstrated in 4/7 patients. There was corresponding myocardial edema in 3/7 patients. B*: LGE was present in a nonischemic pattern consistent with myocarditis in all four patients. Both native T1 and T2 were elevated in the regions with LGE, consistent with an acute injury, except in 1 patient who did not have a T2 map acquired. C*: normal biventricular volumes, morphology, and systolic function. However, there were signs of myocardial fibrosis, hyperemia, and a small pericardial effusion consistent with myopericarditis. D*: showed normal left ventricular function. Short-axis and four-chamber long-axis post-contrast inversion recovery images showed subepicardial LGE in the anterolateral wall of the mid and apical LV. Short-axis native T1 and T2 maps showed corresponding increased T1 (1450–1550 ms; normal: 1100–1300) and T2 (54–60 ms; normal: 40–50) signal intensity, respectively. Measured T1 and T2 values of the normal intraventricular septum were 1200–1300 and 43–46 ms, respectively. E*: Cine images revealed normal left ventricular size and function. Short-axis post-contrast images showed subepicardial enhancement in the inferolateral wall at the base. T1 and T2 maps showed corresponding elevated values of 1200 ms for T1 (normal 950–1050 ms), as opposed to 950–980 for the septal wall, and 59–63 ms for T2 (normal 45–55 ms) as opposed to 44–48 ms for the septal wall. NSAIDS: non-steroidal anti-inflammatory drugs; ACE: angiotensin-converting enzyme; IVIG: intravenous immunoglobulins; SLED HD: sustained slow efficiency dialysis hemodialysis; ECMO: extracorporeal membrane oxygenation

Study	Vaccine type	Age	Gender	ECG	Troponin	Symptoms	Echocardiography	Cardiac MRI	Treatment	Course
Habib et al. [8]	BNT16B2	37	Male	Mild ST elevation	Elevated	Chest pain	Normal	Lake criteria	NSAIDS	Mild
Tailor et al. [9]	mRNA1273	44	Male	Lateral precordial ST elevation	Elevated	Chest pain, dyspnea	EF 40%, global hypokinesia	Lake criteria	Colchicine, ACE inhibitor, B blockers	Mild
Choi et al. [10]	BNT16B2	22	Male	Ventricular fibrillation	-	Chest pain, unconscious	-	-	CPR, 2 hours	Death
Albert et al. [11]	mRNA1237	24	Male	Sinus	Elevated	Chest pain	Normal	Lake criteria	-	Mild
Abu Mouch et al. [12]	BNT16B2	23	Males	-	-	Chest discomfort	-	Lake criteria	-	Mild
D'Angelo et al. [13]	BNT16B2	30	Male	Subtle ST elevation	Elevated	Fever, chest pain, dyspnea	-	Lake criteria	Bisoprolol, aspirin, prednisolone	Mild
Rosner et al. [14]	BNT16B2-mRNA1237	25	Males	ST elevation, PR depression	Elevated	Intermittent chest	EF 50-59%	A*	Bisoprolol, anti-inflammatory	Mild
Kim et al. [15]	BNT16B2-mRNA1237	25	3 Males, 1 Female	ST elevation, PR depression	Elevated	Chest pain	-	B*	NSAIDS, colchicine, prednisolone	Mild
McLean and Johnson [16]	BNT16B2	16	Male	Diffuse ST elevation	Elevated	Chest pain	-	C*	IVIG, ibuprofen	Severe
Mansour et al. [17]	mRNA1237	25	Male, Female	Diffuse ST elevation	Elevated	Chest pain	Normal	D*E*	Metoprolol	Mild
García et al. [18]	BNT16B2	39	Male	Narrow QRS, diffuse ST elevation	Elevated	Chest, interscapular pain	Normal	F*	Anti-inflammatory	Mild
Montgomery et al. [19]	mRNA1237	24	Males	Abnormal findings	Elevated	Chest pain	Reduced EF in 4/23 patients	Abnormal in 8/23 patients	-	Mild
Deb et al. [20]	mRNA1237	67	Male	Sinus tachycardia	Elevated	Fever, dyspnea	EF 50-54%	-	Furosemide, BiPAP, antibiotics	Mild
Watkins et al. [21]	BNT16B2	20	Male	Diffuse ST elevation	Elevated	Chest pain	EF 59%	Lake criteria	Colchicine, NSAIDS, B blockers	Mild
Nevet [4]	BNT16B2	24	Males	Diffuse ST elevation	Elevated	Chest pain, fever	Normal	Lake criteria	Colchicine, NSAIDS	Mild
Muthukumar et al. [22]	mRNA1273	52	Male	Sinus rhythm, left axis deviation	Elevated	Chest pain	Normal	Lake criteria	Lisinopril, B blockers	Mild
Khogali and Abdelrahman [23]	mRNA1273	29	Female	Diffuse ST elevation	Elevated	Headache, diarrhea	Normal	-	Pericardiocentesis, inotropes, SLED HD	Mild
Williams et al. [24]	mRNA1273	34	Male	Lateral ST elevation, PR depression	Elevated	Chest pain, dyspnea	EF 43%	Lake criteria	Colchicine, aspirin, B blockers, rifampicin	Mild
Abbate et al. [25]	BNT16B2	34	Male, female	Diffuse ST elevation	-	Nausea, vomiting, chest pain, fever	EF 15-20%	Lake criteria	ICU, ECMO	Death

Also, a recent retrospective cohort study showed that among more than 2.5 million vaccinated candidates who were 16 years or older, 54 cases reviewed met the criteria for myocarditis. Incidence found to be 2.13 cases/100,000 (95% CI, 1.56 to 2.70). The most increased incidence was noted in male patients between the ages of 16 and 29. Seventy-six percent of myocarditis cases were described as mild, and 22% as intermediate; one case developed cardiogenic shock. After a median follow-up of 83 days from myocarditis onset, one patient was readmitted to the hospital, and one died of an unknown cause after discharge [26].

A diagnosis of myocarditis in these cases was made based on clinical presentation, laboratory tests, and radiologic findings. The most common clinical presentation included chest pain, fever, malaise, and shortness of breath. Commonly associated abnormalities included elevated troponin levels, ECG changes with ST/T wave abnormalities, and echocardiography showed wall motion abnormalities. The clinical similarities in the presentations of these patients, their recent vaccination with an mRNA-based COVID-19 vaccine, and the lack of alternative etiologies for acute myocarditis suggest an association with immunization. Myocarditis or pericarditis was not detected in the clinical trials for these vaccines; however, any association may be too rare for recognition in a clinical trial enrolling less than several hundred thousand participants. Vaccine-associated myocarditis is an uncommon commodity described in the smallpox vaccine [27]. Among around the 416 600 adults receiving live measles, mumps, and rubella; varicella; oral polio; or yellow fever viral vaccinations in the Vaccine Safety Datalink, there were no patients with myocarditis in the 42 days following immunization [28].

The recent data about the risk of myocarditis following immunization with mRNA vaccines from the Israeli Ministry of Health recently posted describing 121 myocarditis cases occurring within 30 days of the second booster dose of mRNA vaccine among 5,049,424 persons, implying a crude incidence rate of approximately 24 cases per million following the second dose in this subset of their vaccinated population [29]. The pattern of clinical presentation, rapid recovery, and absence of evidence of other causes support the diagnosis of hypersensitivity myocarditis. Histology cannot be defined without a myocardial biopsy, but the clinical course points toward eosinophilic hypersensitivity myocarditis, as illustrated in other drug-associated and vaccine-associated myocarditis [30-32].

Mortality due to myocarditis induced by mRNA vaccination is extremely rare; in one study, an autopsy pathology report reported a diffuse inflammatory infiltration, with neutrophil and histiocyte predominance, was observed within the myocardium. Notably, the inflammatory infiltrates were dominant in the atria and around the sinoatrial (SA) and atrioventricular (AV) nodes, whereas the ventricular area displayed minimal or no inflammatory cells [10].

This association with vaccination is rare; the more accurate data on prevalence will be available soon with the results of reporting system of vaccine adverse effects; definitely, the outcomes of immunization in preventing severe morbidity favor continued COVID-19 vaccination. The CDC report recommends that despite a higher risk of vaccine-associated myocarditis, COVID-19 immunization appears to be associated with significantly fewer adverse outcomes than COVID-19 infection among all age groups [33].

## Conclusions

Although more frequent in young male patients, the data available indicate that the risk of acute myocarditis related to COVID-19 immunization is relatively low. In addition, vaccine-related myocarditis typically resolves on its own. These findings should reassure medical professionals and patients that SARS-CoV-2 mRNA vaccination benefit-risk analysis demonstrates a favorable balance for vaccination across all age and sex groups.

## References

[1] Caforio AL, Pankuweit S, Arbustini E (2013). Current state of knowledge on aetiology, diagnosis, management, and therapy of myocarditis: a position statement of the European Society of Cardiology Working Group on Myocardial and Pericardial Diseases. Eur Heart J.

[2] Inciardi RM, Lupi L, Zaccone G (2020). Cardiac involvement in a patient with coronavirus disease 2019 (COVID-19). JAMA Cardiol.

[3] Wise J (2021). Covid-19: should we be worried about reports of myocarditis and pericarditis after mRNA vaccines?. BMJ.

[4] Nevet A (2021). Acute myocarditis associated with anti-COVID-19 vaccination. Clin Exp Vaccine Res.

[5] Bozkurt B, Kamat I, Hotez PJ (2021). Myocarditis with COVID-19 mRNA vaccines. Circulation.

[6] Das BB, Moskowitz WB, Taylor MB, Palmer A (2021). Myocarditis and pericarditis following mRNA COVID-19 vaccination: what do we know so far?. Children.

[7] Karatolios K, Pankuweit S, Maisch B (2007). Diagnosis and treatment of myocarditis: the role of endomyocardial biopsy. Curr Treat Options Cardiovasc Med.

[8] Habib MB, Hamamyh T, Elyas A, Altermanini M, Elhassan M (2021). Acute myocarditis following administration of BNT162b2 vaccine. IDCases.

[9] Tailor PD, Feighery AM, El-Sabawi B, Prasad A (2021). Case report: acute myocarditis following the second dose of mRNA-1273 SARS-CoV-2 vaccine. Eur Heart J Case Rep.

[10] Choi S, Lee S, Seo JW (2021). Myocarditis-induced sudden death after BNT162b2 mRNA COVID-19 vaccination in Korea: case report focusing on histopathological findings. J Korean Med Sci.

[11] Albert E, Aurigemma G, Saucedo J, Gerson DS (2021). Myocarditis following COVID-19 vaccination. Radiol Case Rep.

[12] Abu Mouch S, Roguin A, Hellou E (2021). Myocarditis following COVID-19 mRNA vaccination. Vaccine.

[13] D'Angelo T, Cattafi A, Carerj ML (2021). Myocarditis after SARS-CoV-2 vaccination: a vaccine-induced reaction?. Can J Cardiol.

[14] Rosner CM, Genovese L, Tehrani BN (2021). Myocarditis temporally associated with COVID-19 vaccination. Circulation.

[15] Kim HW, Jenista ER, Wendell DC (2021). Patients with acute myocarditis following mRNA COVID-19 vaccination. JAMA Cardiol.

[16] McLean K, Johnson TJ (2021). Myopericarditis in a previously healthy adolescent male following COVID-19 vaccination: a case report. Acad Emerg Med.

[17] Mansour J, Short RG, Bhalla S, Woodard PK, Verma A, Robinson X, Raptis DA (2021). Acute myocarditis after a second dose of the mRNA COVID-19 vaccine: a report of two cases. Clin Imaging.

[18] García JB, Ortega PP, Fernández JAB, León AC, Burgos LR, Dorta EC (2021). Acute myocarditis after administration of the BNT162b2 vaccine against COVID-19. Rev Esp Cardiol (Engl Ed).

[19] Montgomery J, Ryan M, Engler R (2021). Myocarditis following immunization with mRNA COVID-19 vaccines in members of the US Military. JAMA Cardiol.

[20] Deb A, Abdelmalek J, Iwuji K, Nugent K (2021). Acute myocardial injury following COVID-19 vaccination: a case report and review of current evidence from vaccine adverse events reporting system database. J Prim Care Community Health.

[21] Watkins K, Griffin G, Septaric K, Simon EL (2021). Myocarditis after BNT162b2 vaccination in a healthy male. Am J Emerg Med.

[22] Muthukumar A, Narasimhan M, Li QZ (2021). In-depth evaluation of a case of presumed myocarditis after the second dose of COVID-19 mRNA vaccine. Circulation.

[23] Khogali F, Abdelrahman R (2021). Unusual presentation of acute perimyocarditis following SARS-COV-2 mRNA-1237 Moderna vaccination. Cureus.

[24] Williams CB, Choi JI, Hosseini F, Roberts J, Ramanathan K, Ong K (2021). Acute myocarditis following mRNA-1273 SARS-CoV-2 vaccination. CJC Open.

[25] Abbate A, Gavin J, Madanchi N (2021). Fulminant myocarditis and systemic hyperinflammation temporally associated with BNT162b2 mRNA COVID-19 vaccination in two patients. Int J Cardiol.

[26] Witberg G, Barda N, Hoss S (2021). Myocarditis after Covid-19 vaccination in a large health care organization. N Engl J Med.

[27] Eckart RE, Love SS, Atwood JE (2004). Incidence and follow-up of inflammatory cardiac complications after smallpox vaccination. J Am Coll Cardiol.

[28] Kuntz J, Crane B, Weinmann S, Naleway AL (2018). Myocarditis and pericarditis are rare following live viral vaccinations in adults. Vaccine.

[29] Ludwig A, Lucero-Obusan C, Schirmer P, Winston C, Holodniy M (2015). Acute cardiac injury events ≤30 days after laboratory-confirmed influenza virus infection among U.S. veterans, 2010-2012. BMC Cardiovasc Disord.

[30] Tschöpe C, Ammirati E, Bozkurt B (2021). Myocarditis and inflammatory cardiomyopathy: current evidence and future directions. Nat Rev Cardiol.

[31] Engler RJ, Nelson MR, Collins LC Jr (2015). A prospective study of the incidence of myocarditis/pericarditis and new onset cardiac symptoms following smallpox and influenza vaccination. PLoS One.

[32] Aslan I, Fischer M, Laser KT, Haas NA (2013). Eosinophilic myocarditis in an adolescent: a case report and review of the literature. Cardiol Young.

[33] Saif Abu Mouchab, Ariel Roguinbc, Elias Helloubc, etc etc (2022). Myocarditis and Pericarditis After mRNA COVID-19 Vaccination | CDC. science direct.

